# Psychological Support System for Hospital Workers During the Covid-19 Outbreak: Rapid Design and Implementation of the Covid-Psy Hotline

**DOI:** 10.3389/fpsyt.2020.00511

**Published:** 2020-05-28

**Authors:** Pierre A. Geoffroy, Véronique Le Goanvic, Olivier Sabbagh, Charlotte Richoux, Aviv Weinstein, Geoffrey Dufayet, Michel Lejoyeux

**Affiliations:** ^1^Department of Psychiatry and Addictive Medicine, Assistance Publique-Hôpitaux de Paris (AP-HP), University Hospital Bichat, Paris, France; ^2^Université de Paris, NeuroDiderot, Inserm, Paris, France; ^3^Sector of Psychiatry, G.H.U. Paris et Neuroscience, Paris, France; ^4^Ariel University, Ariel, Israël; ^5^Université de Paris, Paris, France

**Keywords:** healthcare workers, medical workers, psychological intervention, mental health, psychiatry, psychology

## Abstract

**Background:**

The worldwide coronavirus outbreak has put hospital workers under extreme stress with possible mental health problems. In this context, we decided to rapidly design and implement a psychological support system for all hospital workers in Paris during the Covid-19 outbreak.

**Methods:**

We built a hotline in 3 days using the following steps: 1) official mandate, 2) request for the creation of hotline numbers, 3) formulation of psychological intervention materials and policies, 4) call for volunteer certified psychologists, 5) call for volunteer certified psychiatrists in case of psychiatric cases, 6) creation of an anonymous and protected database, and 7) communication and regular reminders about the existence of the hotline for hospital workers.

**Results:**

After the first 26 days, we received 149 calls with a mean of 5.73 calls/day (SD=3.22). The average call duration was 18.5 min (min=1; max=65min; SD=14.7), and mostly women (86%) called. The mean age was 32.7 years old (SD=11.0). Calls from hospital workers were from all professions; though mostly represented by frontline healthcare workers, non-frontline departments also called (total of 44 departments). Reasons for calling were anxiety symptoms (n=73, 49%), request for hotline information (n=31, 20.8%), worries about Covid-19 (n=23, 15.44%), exhaustion (n=17, 11.41%), trauma reactivation (n=10, 6.11%), insomnia (n=9, 6.0%), anger (n=8, 5.37%), depressive (n=6, 4.02%), and psychotic symptoms (n=3, 2.01%). Regarding referrals, 105 (70.47%) of them were referred to psychosocial, Covid, and general support.

**Conclusions:**

This psychological support system can be easily duplicated and seems to benefit all hospital professions that all appeared psychologically affected.

## Introduction

The worldwide coronavirus outbreak of pneumonia emerged in December 2019 in Wuhan in the Hubei province of China (1). In January 2020, Chinese scientists isolated a novel coronavirus with the rapid development of RT-PCR diagnostic tests specific for this 2019 novel coronavirus (2019-nCoV; also more commonly named Covid-19) (1). Acute respiratory distress syndrome may occur for 42% of patients with Covid-19 with a mortality rate above 50% for those patients (2). France had the first individuals affected by January 24 and, to date, ranks seventh in terms of the number of confirmed cases of Covid-19 infection. A nationwide lockdown began on March 17 and was extended until May 11 to better contain the spread of the Covid-19 and help overwhelmed hospitals (3). This unprecedented situation has put healthcare workers under extreme stress with possible moral injuries or mental health problems (4). It very quickly became clear that we needed to develop a psychological support system for hospital workers, and this was requested by the direction of the Assistance Publique – Hôpitaux de Paris (AP-HP).

Indeed, experiences from China were that healthcare workers in Wuhan have been facing enormous pressure and overwork, thus leading to mental health problems, including stress, anxiety, depressive symptoms, insomnia, denial, anger, and fear (5). The observations of Chinese colleagues were that mental health problems affected both the professional functioning and overall well-being of healthcare workers (5). In addition, first observations also reported that frontline medical workers may present with vicarious traumatization (negative transformation in the helper with psychological abnormalities that are derived from sympathy for survivors of a trauma, which causes serious physical and mental distress—even mental breakdown), and there is an increased risk for non-frontline hospital workers (6). In a very recent cross-sectional survey in 1,257 healthcare workers, up to 71.5% of them reported distress, 50.4% symptoms of depression, 44.6% anxiety, and 34% insomnia (7). In this context, in order to prevent or early intervene in case of mental health problems, we decided to rapidly design and implement a psychological support system for all hospital workers during the Covid-19 outbreak in Paris. This paper aims to 1) present the methods for implementing such psychosocial support system we called the Covid-Psy hotline and 2) characterize first calls and reasons for the call.

## Methods

### Implementing the Hotline

**Table 1** summarizes the different steps for developing a psychosocial support system for healthcare workers.

**Table 1:** Rapid design and implementation of a psychosocial support system for healthcare workers: Steps for implementing the Covid-Psy hotline in 3 days.

Step 1	Official mandate from the authorities to lead and develop the project (an essential step for its successful promotion and unifying dimension).
Step 2	Request for the creation of hotline numbers with possible transfer to psychologists' personal mobile phones (this step may take some time and may need several lines to be provided).
Step 3	Formulation of psychological intervention materials and policies (including a list with numbers and addresses of possible psychological and psychiatric referral locations)
Step 4	Call for volunteer certified psychologists to help on this hotline (creation of a list of volunteers with personal contact details, and a messaging group to facilitate communications between volunteers)
Step 5	Call for volunteer certified psychiatrists in case of psychiatric cases (creation of a list of volunteers with personal contact details)
Step 6	Creation of an anonymous and protected database (in order to be able to improve and evolve the support)
Step 7	Communication and regular reminders of the existence about the hotline for hospital workers

These steps have been made in only three days, the hotline being opened and active the third day. The first step was to get an official mandate, which was granted from the direction of the Assistance Publique – Hôpitaux de Paris (AP-HP) who mandated ML to propose an anonymous and psychological support for all its workers, 24/7. This step was a major one because it guarantees the feasibility of the project, its successful promotion, and its unifying dimension. The second step was the creation of hotline numbers with possible transfer to psychologists' personal mobile phones. According to our experience, this step should be launched as soon as possible since it may take some time due to technical resources. In addition, we decided to ask for three numbers to be in capacity to respond to all calls (and so up to three calls at the same moment). The third step was the formulation of the psychological intervention materials and policies, which include a list with numbers and addresses of possible psychological and psychiatric referral locations. The fourth step was a call for certified psychologists to volunteer help on this hotline. VLG, who was the coordinating psychologist, created a list of volunteers with personal contact details and a messaging group to facilitate communications between volunteers. The fifth step was carried out by ML: certified psychiatrists were called upon to volunteer in case of psychiatric cases, and a list of volunteers and their personal contact details was created. Then, for the sixth step, PAG created an anonymous and protected database in order to be able to improve and assess the support (such as the change for 2 work schedules, 8am–7pm and 7pm–8am, to 3 work schedules, 8am–2pm, 2pm–8pm, and 8pm–8am; pairing during each work schedule except at night, as few calls have been made so far at night). The project was also submitted to the Research Ethics Board of our hospital. The last step is a large communication about this hotline (emails, posters, newsletters, Twitter, Facebook, etc.) and regular reminders of the existence of the hotline for hospital workers (and not only healthcare professionals since more indirect professionals are also dealing with difficulties).

### Interventions

Briefly, the psychological assistance hotline team was composed of certified psychologist volunteers. VLG was responsible for formulating psychological intervention materials and policies (guidelines for the intervention, hotline organization and technical functioning, and lists of volunteers and referrals). VLG certified volunteers through a 30-min session by phone on brief crisis intervention with rapid assessment and crisis resolution or referrals (see the types of assistance provided below). No specific crisis intervention models or algorithms were used (8). At the same time, to provide a secure base for this internal AP-HP teams, OS has built up a reserve of volunteer clinicians who can be mobilized. Psychologists can consult with a psychiatrist (ML or PG) to discuss the cases and then call the original caller back to propose a referral or to organize a further meeting or call with the psychiatrist. ML and PG provided supervision for situations requiring a psychiatric opinion. A case discussion was proposed twice a week by external psychologists.

The types of assistance provided by the hotline:

- The reason for the call expressed by the hospital worker for an average of about 20 minutes
- Identification of symptoms
- Proposal of responses according to guidelines and to symptoms identified:
• Short individual response, if sufficient, without particular referrals
• Referral to other psychosocial supports, including cognitive behavioral therapies or psychotherapies more focused on trauma
• Referral to medical specialized additional expertise, including psychiatric consultation

#### First-Line Intervention

First-line volunteers were certified psychologists from university hospitals at AP-HP with expansion of this recruitment on March 23 to external psychologists to reinforce the team, which now consists of about 30 volunteers. VG provided full-time work for three weeks to coordinate the hotline, and they were assisted by a colleague (GD) on a part-time basis. Over the following weeks, VG dedicated 80% of her time to assure the current functioning and to manage the technical problems. The extended list of 30 voluntary psychologists allowed the maintenance of their current clinical activities.

#### Second-Line Intervention

Second-line interventions available according to situation and as called by the volunteer include

- Possible call to the psychiatrist working 24/7 in each emergency room of each AP-HP hospitals
- A specialized trauma telephone platform at the Hôtel-Dieu (Help-line) available 9:30am–6 pm from Monday to Friday
- Links with the medical-psychological emergency cells (CUMP, Plan Blanc Psy, Dr Abgrall)
- Orientation towards other local psychological support
- Occupational health and safety department at each of the AP-HP sites with possible Covid-19 screening on appointments
- Infectious Disease department in case of a question directly related to the Covid involving a “medical” answer not provided by psychiatrists

#### Backup of Psychologists and Psychiatrists Includes

- Backup team made up of volunteer psychologists and psychiatrists in case of increased need

### Data

The following data are collected and entered online or offline, using a predefined scoring grid to simplify statistical analysis, on an Excel file on a secure AP-HP workstation:

- Date of the call
- Gender
- Age
- Hospital
- Service
- Profession
- Call time
- Call duration
- Reasons for the call
- Psychiatric history
- Orientation
- Free text to detail the problem/concern

The Data Protection and Security Compliance Diagnostic Report was made by the AP-HP Data Protection Office under number BPD2018DIA008.

### Population

This psychological support was accessible for all hospital workers (healthcare and non-healthcare workers) from the Assistance Publique – Hôpitaux de Paris (AP-HP), which is a regional group of 39 hospitals in Ile de France, France. The 39 hospitals are organized and united in six university hospital groups, as detailed in **Figure S1**. AP-HP employs more than 100,000 professionals, including nearly 1,300 doctors, 3,600 residents, and more than 52,000 nursing, paramedical, and socio-educational staff.

### Statistics

Descriptive analyses were made using Excel and an R software package provided by the R Foundation for Statistical Computing. Qualitative variables were expressed as frequencies and percentages. Quantitative variables were expressed as means and standard deviation (SD) with min and max values.

## Results

### Hotline Activity

**Figure 1:**
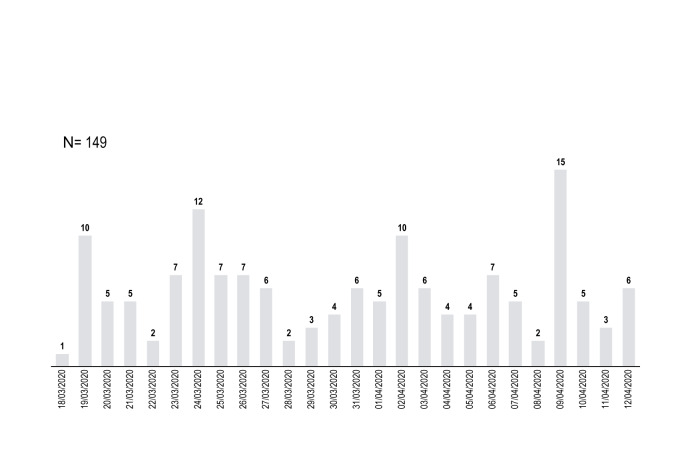
Distribution of the daily calls [in number of calls] to the hotline Covid-Psy for a total of 149 calls in 26 days.

After the first 26 days of the hotline activity, we received 149 calls. **Figure 1** summarizes the distribution of calls per day, showing a large variability of calls number per day ranging from minimum 1 to maximum 15 calls per day with a mean of 5.73 calls/day (SD=3.22).

**Figure 2:**
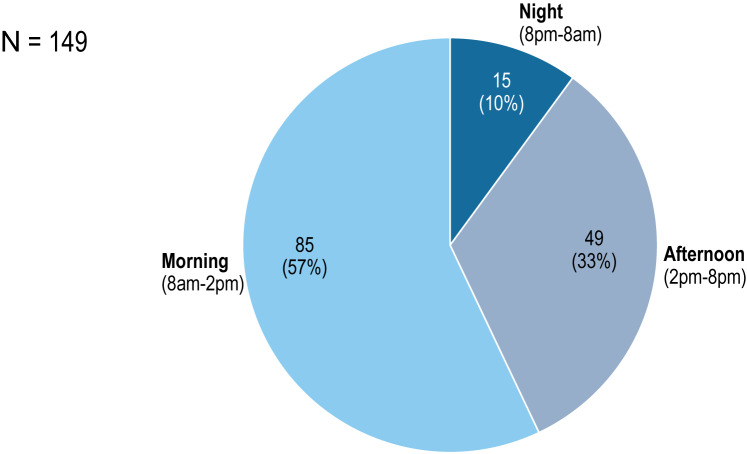
Hours of call to the Covid-Psy hotline [number of calls and %].

The average duration of a call to the hotline was 18.5 min (min=1; max=65min; SD=14.7). Calls were mainly during the morning (between 8am until 2pm) with 85 calls (57%), then during the afternoon (2pm-8pm) with 49 calls (33%), and lastly during the night (8pm-8am) with 15 (10%) of calls (**Figure 2**).

### Population

Mostly women (86%) called the hotline. The mean age of callers was 32.7 years old (min=19; max=56min; SD=11.0).

**Figure 3:**
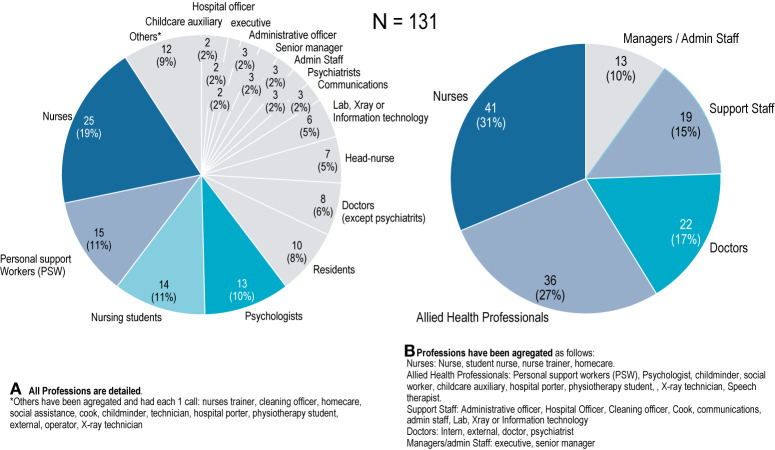
Hospital professions calling to the hotline Covid-Psy [number of professions].

Hospital workers calling were from all professions, including mostly registered nurses (n=25; 19%), personal support workers (PSW) (n=15; 11%), nursing students (n=14; 11%); psychologists (n=13; 10%), residents (n=10; 8%), senior doctors (n=8; 6%), head-nurses (n=7; 5%), Lab, X-Ray, or Information technology (n=6; 5%), senior head-nurses (n=3; 2%), administrative staff (n=3; 2%), communications (n=3; 2%), psychiatrists (n=3; 2%), and administrative officers (n=3; 2%). All professions are reported in **Figure 3**.

The analysis of the different departments who called the Covid-Psy hotline showed that 44 different hospital departments called the hotline Covid-Psy, highlighting that the most exposed and frontline departments called the most: emergency room (8% of total calls), nursing school (7%), intensive care units and Covid specialized units (6%), and the Infectious Disease department (6%). Interestingly, numerous other non front-line departments appeared affected, including non-health-care workers, as summarized in **Table 2**. All hospitals from the AP-HP called the hotline, and dates are summarized in **Figure S1**.

**Table 2:** Hospital departments calling the hotline Covid-Psy [number of call and % of calls].

Hospital departments (N= 44)	Number of calls (N=100) (per department)	% of total calls
Emergency Room	8	8
Nursing school	7	7
intensive care units, Covid specialized units	6	6
Infectious disease department, Rehab, support services	5	5
Community doctors	4	4
Surgery, Geriatrics, Hepato-gastro, Cardiology, Presse department	3	3
Maternity, Radiology, Functional exploration, Outpatient care, Occupational health and safety department, Anesthesia, Oncology, Hematology, Administrative office	2	2
Lab, Addictions, Internal Medicine, Biochemistry, Physical medicine, Union Service, Neonatology, Crib, Nephrology, Finance department, Neurology, Immunology, Oto-rhino laryngology, Urology, Orthopedics, Operating, Pneumology, Kitchen, Back-up department, Staff service, Admissions	1	1

### Reasons for the Call

**Figure 4:**
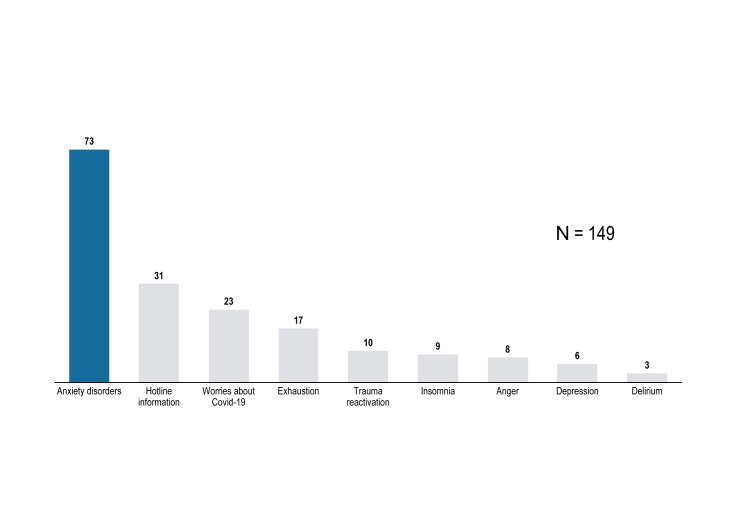
Occurrences of the reason for calling the hotline Covid-Psy [number].

Anxiety symptoms were the first cause for hospital workers to call the hotline and affected 73 (49%) of them. Other reasons were requests for hotline information (n=31, 20.8%), worries about Covid-19 (n=23, 15.44%), exhaustion (n=17, 11.41%), trauma reactivation (n=10, 6.11%), insomnia (n=9, 6.0%), anger (n=8, 5.37%), depressive symptoms (n=6, 4.02%), and psychotic symptoms (n=3, 2.01%). **Figure 4** summarizes occurrences of all different reasons for calling the hotline Covid-Psy.

### Referrals

**Figure 5:**
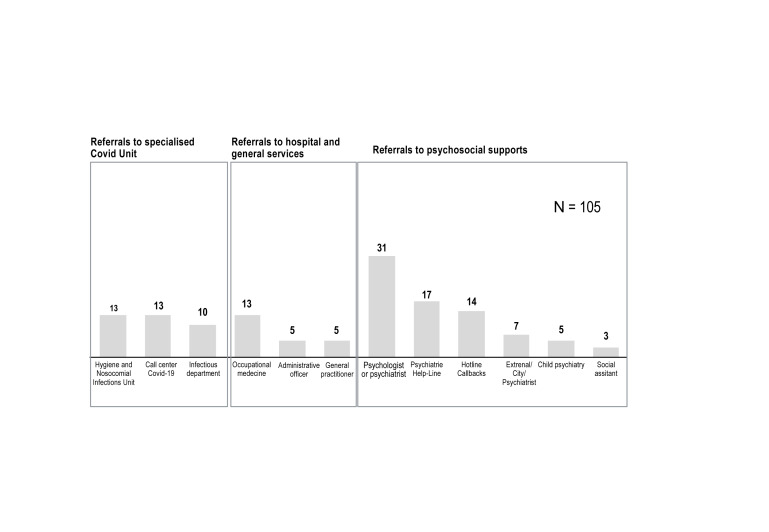
Referrals for hospital workers calling the Covid-Psy hotline [number of referrals].

Regarding referrals proposed to hospital workers who called the hotline (**Figure 5**), 105 (70.47%) of them were referred to psychosocial, Covid, and general support. Among them, 29.5% (31/105) were referred to a psychologist or psychiatrist, 16.2% (17/105) were referred to the psychiatry helpline, 13.3% (14/105) preferred calling the hotline back, 6.6% (7/105) were referred to an external/city psychiatrist, 6.6% (7/105) had their child referred to a child psychiatrist, and 2.9% (3/105) were referred to a social worker.

Regarding referrals to specialized Covid units, 12.4% (13/105) were referred to the Hygiene and Nosocomial Infections Unit, 9.5% (10/105) to the infectious department, and 12.4% (13/105) to the Call Center Information for Covid-19 (**Figure 5**).

Regarding referrals to hospital and general services, 12.4% (13/105) were referred to the occupational medicine, 4.8% (5/105) to the administrative office, and 4.8% (5/105) to a general practitioner (**Figure 5**).

## Discussion

At present, this is the first report of a psychological hotline design and implementation for the stress induced by the Covid-19, which can be replicated in only 3 days in other hospitals and countries. We found that all hospital professions and departments have workers who are experiencing psychological distress. We were surprised by the numerous non-frontline workers that were affected, leading us to intervene directly in these departments, including admissions, mortuary, informatics, radiology, hospital porter, technical, mail service, etc. Of note, we found a high prevalence of new psychiatric symptoms manifesting in hospital workers during this Covid-19 outbreak, which could have been underestimated because of the absence of standardized evaluation. This explains the high numbers of referrals, especially regarding psychosocial supports.

The stress reported by callers might be summarize in three main dimensions with 1) the “direct Covid-19 stress”—stress of being contaminated, of dying, and of contaminating loved ones; 2) the “social stress”—numerous hospital workers met difficulties at home feeling either isolated, poorly understood, or suffering from intra-family tensions; and 3) the “work-related stress”—with numerous changes at work, loss of routines, and new procedures and materials. These psychological and occupational impacts are similar to those observed during the 2003 SARS Outbreak (9–11), and their understanding is important in planning for future outbreaks of emerging infectious diseases.

A post-hoc examination of the free text emphasized that profiles of calls changed over time with mostly mild symptoms at the beginning, such as anxiety, “stress,” and worries about the Covid-19; the last week, we noticed an increase of more severe problems, such as anxiety and depressive symptoms, sleep disorders, exhaustion (or “burn-out”), and psychosis. We also observed an increase in reactivations of previous traumas. We also noted the marked fragility and difficulties of the new healthcare workers who arrived as reinforcements to already constituted teams. Difficulties met were the reorganization of care habits, the unfamiliarity of the department's functioning, the lack of training/information, and the social isolation, all this in spite of the welcoming the reinforcements received by the constituted team and the acquisition of new equipment.

This psychological support is complementary to other supports we have detailed in the methods and as observed in the referrals. As in Wuhan (5), hospitals in Paris benefited also from local psychological intervention teams with psychologists (face-to-face meetings and on-site outreach mobile team the week-end) and the psychiatry team, who are mainly psychiatrists, participating in clinical psychological intervention for healthcare workers and patients in the hospital. Lastly, other psychological assistance hotline teams exist, such as the specialized trauma telephone platform at the Hôtel-Dieu (Help-line), and this allowed to propose a complete and complementary support to hospital workers.

Some limitations should be acknowledged. Because the main objective of this hotline was the provision of anonymous psychological support with brief interventions, we have no feedback on caller satisfaction or the follow-up for the referrals. Moreover, it was not possible to estimate the prevalence of these disorders since other possible local supports may exist, and there were no questions made to the caller as to whether they had tried to reach their own local support team. In addition, the hotline cannot make a formal diagnosis. Finally, we should be aware of the opportunity cost of using staff working in other areas to set up the psychological support, which could be limited by recruiting many volunteers.

## Conclusion

This psychological support system was rapidly designed and implemented in 3 days and can thus be easily duplicated. It seems to benefit to all hospital professions that all appeared psychologically affected. Significant psychological repercussions included psychiatric disorders like trauma reactivation, anxiety, depressive, insomnia, and psychotic symptoms. Mandatory factors for the implementation of the hotline include a clear mandate, the adequate and appropriate human resources (volunteers), a functional technology platform, ensuring anonymity, as well as a clear communication plan (sending regular reminders about the 24/7 hotline). Finally, these observations emphasize the need for Health Authorities to be informed of the psychological impact of a pandemic on the welfare of their employees and their workplace performance in order to offer the psychological support and the help needed.

## Data Availability Statement

The raw data supporting the conclusions of this article will be made available by the authors, without undue reservation.

## Ethics Statement

The studies involving human participants were reviewed and approved by CEERB Paris Nord. Written informed consent for participation was not required for this study in accordance with the national legislation and the institutional requirements.

## Author Contributions

PG, VG, and ML designed the study. VG coordinated the hotline and was responsible for formulating psychological intervention materials and rules. ML and PG provided supervision for situations requiring a psychiatric opinion. PG wrote the first draft of the manuscript. PG, VG, and OS made the statistics and figures. PG made the table. PG, VG, OS, CR, AW, GD, and ML participated in the writing and approved the submitted version of the work.

## Conflict of Interest

The authors declare that the research was conducted in the absence of any commercial or financial relationships that could be construed as a potential conflict of interest.
