# Supplementary material for: Psychological Support System for Hospital Workers During the Covid-19 Outbreak: Rapid Design and Implementation of the Covid-Psy Hotline
Source: Front Psychiatry. 2020 May 28;11:511. doi: 10.3389/fpsyt.2020.00511 (PMC7326137; doi:10.3389/fpsyt.2020.00511)

Figure S1. Distribution of hospitals calling the Covid-Psy hotline frome the Assistance Publique – Hôpitaux de Paris (APHP) [number of calls and %]

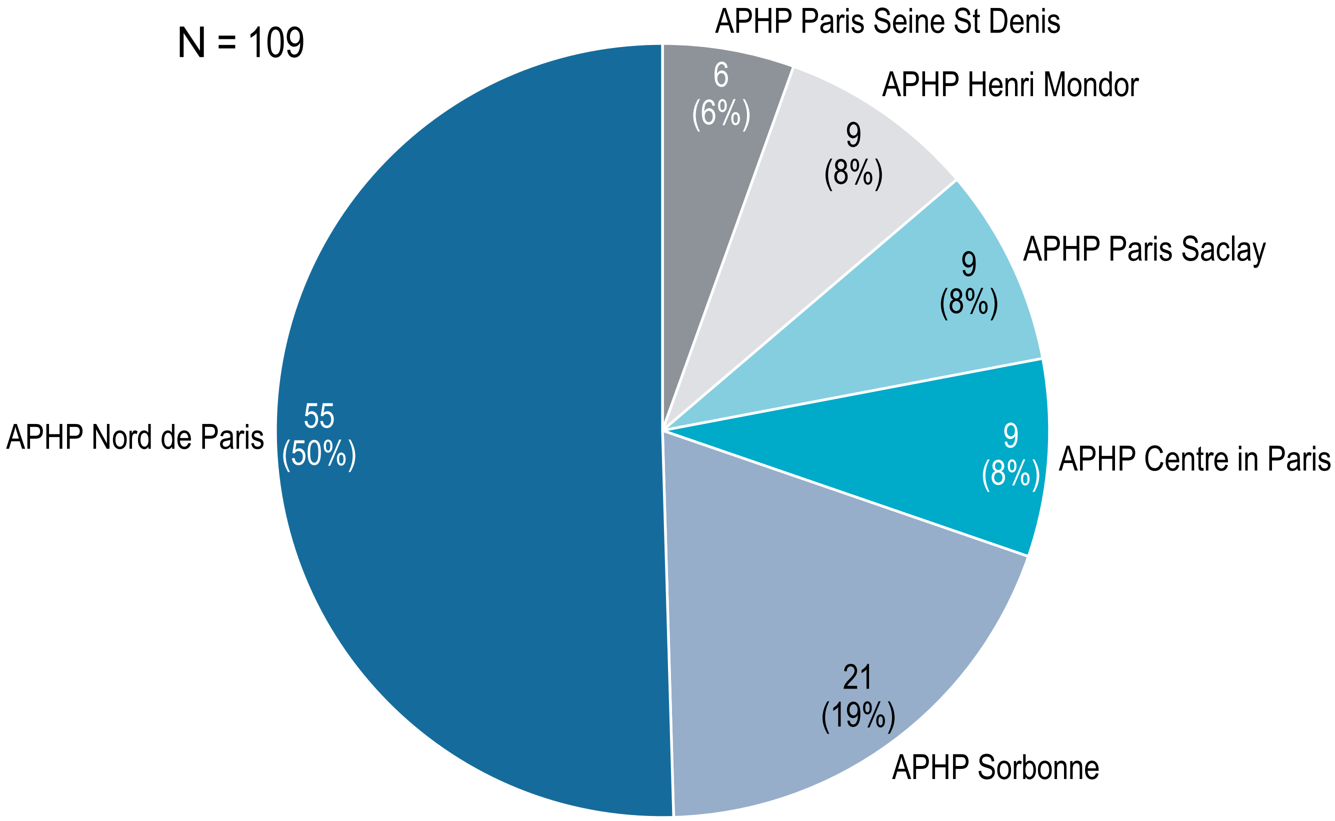

Supplement: Supplementary file 1 [file Image_1.pdf]
